# Discordant Liver Fibrosis Predictors in Virologically Suppressed People Living with HIV without Hepatitis Virus Infection

**DOI:** 10.3390/diagnostics12010014

**Published:** 2021-12-22

**Authors:** Barbara Rossetti, Valentina Borgo, Arianna Emiliozzi, Marta Colaneri, Giacomo Zanelli, Miriana d’Alessandro, Davide Motta, Laura Maiocchi, Francesca Montagnani, Maria Cristina Moioli, Chiara Baiguera, Margherita Sambo, Teresa Chiara Pieri, Pietro Valsecchi, Raffaele Bruno, Massimo Puoti, Massimiliano Fabbiani

**Affiliations:** 1 Infectious and Tropical Diseases Unit, Siena University Hospital, 53100 Siena, Italy; valentina.borgo1989@gmail.com (V.B.); giacomo.zanelli1971@gmail.com (G.Z.); francesca.montagnani@unisi.it (F.M.); massimiliano.fabbiani@gmail.com (M.F.); 2 Department of Medical Biotechnologies, University of Siena, 53100 Siena, Italy; ariannaemiliozzi@gmail.com; 3 Infectious Diseases I Unit, I.R.C.C.S. Policlinico San Matteo Foundation, 27100 Pavia, Italy; marta.colaneri@gmail.com (M.C.); L.Maiocchi@smatteo.pv.it (L.M.); sambomargherita@gmail.com (M.S.); teresachiara.pieri01@universitadipavia.it (T.C.P.); pietro.valsecchi01@universitadipavia.it (P.V.); raffaele.bruno@unipv.it (R.B.); 4 Respiratory Diseases and Lung Transplant Unit, Department of Medical and Surgical Sciences and Neurosciences, University of Siena, 53100 Siena, Italy; dalessandro.miriana@gmail.com; 5 Infectious Diseases Unit, Niguarda Hospital, 20162 Milan, Italy; davide.motta@ospedaleniguarda.it (D.M.); mariacristina.moioli@ospedaleniguarda.it (M.C.M.); chiara.baiguera@ospedaleniguarda.it (C.B.); massimo.puoti@ospedaleniguarda.it (M.P.)

**Keywords:** liver fibrosis, HIV, ART

## Abstract

Severe liver fibrosis (LF) is associated with poor long-term liver-related outcomes in people living with HIV (PLWH). The study aimed to explore the prevalence and predictors of LF and the concordance between different non-invasive methods for the estimation of LF in HIV-infected individuals without hepatitis virus infection. We enrolled PLWH with HIV-1-RNA <50 copies/mL for >12 months, excluding individuals with viral hepatitis. LF was assessed by transient elastography (TE) (significant >6.65 kPa), fibrosis-4 (FIB-4) (significant >2.67), and AST-to-platelet ratio index (APRI) (significant >1.5). We included 234 individuals (67% males, median age 49 years, median time from HIV diagnosis 11 years, 38% treated with integrase strand transfer inhibitors). In terms of the TE, 13% had ≥F2 stage; FIB-4 score was >1.5 in 7%; and APRI > 0.5 in 4%. Higher body mass index, diabetes mellitus, detectable baseline HIV-1 RNA and longer atazanavir exposure were associated with higher liver stiffness as per TE. Predictors of higher APRI score were CDC C stage and longer exposure to tenofovir alafenamide, while HBcAb positivity and longer exposure to tenofovir alafenamide were associated to higher FIB-4 scores. Qualitative agreement was poor between FIB-4/TE and between APRI/TE by non-parametric Spearman correlation and kappa statistic. In our study, in the group of PLWH without viral hepatitis, different non-invasive methods were discordant in predicting liver fibrosis.

## 1. Introduction

Human immunodeficiency virus (HIV) infection is one of the most serious public health challenges, affecting approximately 37.6 million people across the globe in 2020, of whom 34 million are adults [1]. As a result of remarkable advances in scientific understanding of HIV and its prevention and treatment, after the introduction of highly active antiretroviral treatment (HAART) back in 1996, the leading causes of morbidity and mortality among people living with HIV (PLWH) in high-income countries have switched from opportunistic infections and AIDS-related neoplasms to non-AIDS related events, especially cardiovascular and liver disorders [2,3,4,5]. Prevalence of liver involvement in PLWH ranges from 4 to 18% according to different studies, while up to 18% of deaths were liver-related in some European real-life cohorts [6,7,8,9,10,11].

Severe liver fibrosis is associated with poor long-term liver-related outcomes and mortality, and PLWH are at higher risk of multifactorial liver injuries than people without HIV [12,13,14,15,16,17,18].

Several studies have confirmed the possible causes contributing to the progression of liver fibrosis in PLWH, in particular, age, coinfections with viral hepatitis and alcohol abuse [19,20,21,22,23,24]. On the other hand, the potential effect of body mass index and the presence of hypertriglyceridemia or diabetes mellitus are debated and inconclusive [22,25]. 

The cornerstone finding in all recent studies is the association between HIV per se and low CD4^+^ cells count with liver fibrosis [26,27,28,29]. HIV primarily infects liver macrophages, but also hepatocytes and hepatic stellate cells, leading to fibrosis by inflammation and necrosis of hepatocyte cells, increased intra-hepatic expression of collagen and enhanced expression of the transforming growth factors [27,28]. Furthermore, HIV directly causes endoplasmic reticulum stress, mitochondrial toxicity and increased oxidative stress, with decreased beta-oxidation of fatty acids and the accumulation of fat in the liver. Indeed, it may also cause a gut barrier dysfunction, leading to high amounts of circulating lipopolysaccharides, responsible of augmented insulin resistance, overproduction of tumor necrosis factor-alpha, triglyceride accumulation, inflammation, non-alcoholic steatohepatitis and lastly liver fibrosis [30,31,32,33,34,35].

Although the START trial showed that the effective control of HIV replication with early ART initiation seems to be protective for liver fibrosis progression, the hepatotoxicity of antiretroviral drugs has been debated and many controversies still exist. Several studies have shown a significant liver injury after treatment with some nucleos(t)ide reverse transcriptase inhibitors (NRTIs), mainly didanosine (DDI) and azidothymidine (AZT) [36,37,38,39]. On the contrary, integrase strand transfer inhibitors (INSTIs) and non-nucleoside reverse transcriptase inhibitors (NNRTIs), with the exception of nevirapine (NVP), are proven to be safe for liver health [40,41,42,43]. Even though protease inhibitors (PIs) cause insulin resistance and lipodystrophy, data from studies regarding hepatotoxicity of this drug class are controversial [44,45,46].

Different non-invasive methods are nowadays available in order to assess the risk of liver damage, mainly including serological panels and radiological test, with transient elastography (TE) being the most extensively and routinely used [47,48].

The majority of these methods have been validated in specific subset of patients. AST to Platelet Ratio Index (APRI) score has been investigated mainly in HCV-infected or HIV-HCV coinfected individuals and in those affected by alcohol-related disease, while fibrosis-4 (FIB-4) score in HCV-infected individuals, in those affected by non-alcoholic fatty liver disease (NAFLD) and among PLWH coinfected with HCV [49,50,51,52,53].

On these bases, the primary outcome of our study was to evaluate the prevalence of significant liver fibrosis in HIV-infected virologically suppressed individuals without hepatitis virus infection by non-invasive methods. Secondary outcomes included the evaluation of the concordance of different non-invasive methods for the estimation of liver fibrosis and the identification of predictors of advanced liver fibrosis in a real-life multicenter cohort.

## 2. Materials and Methods

### 2.1. Study Design and Population

We performed a multicenter retrospective cross-sectional study including PLWH who consecutively underwent liver fibrosis assessment by transient elastography (TE) at three HIV referral centers in Northern and Central Italy (Milan, Pavia and Siena) from January 2014 and February 2020.

We recruited PLWH aged ≥18 years that were HIV-1-infected and treated with antiretroviral drugs from at least 18 months and with virological suppression (defined as HIV-1 RNA <50 copies/mL) from at least 12 months (isolated blips were allowed), with full accessibility to past medical records.

Exclusion criteria were as follows: (i) detectable HCV-RNA or hepatitis B surface antigen positivity; (ii) previous treatment for HCV infection; (iii) significant alcohol intake, defined as daily consumption exceeding 30 g for males and 20 g for females; (iv) evidence of other liver diseases; (v) history of hepatocellular carcinoma or liver transplantation or liver cirrhosis; (vi) contraindications to TE examination (pregnancy, pacemaker insertion); (vii) failure or unreliable measurement of TE examination.

Participants underwent assessment of liver fibrosis by TE and concomitantly by FIB-4 index and APRI score.

Data collection was approved by the local ethics committees and informed consent was obtained from all patients before participation. The study was carried out in accordance with the ethical principles of the Declaration of Helsinki and with the Good Clinical Practice guidelines of the International Conference on Harmonization.

### 2.2. Study Procedures

At the time of enrollment, clinical and anthropometric data were collected including demographic characteristics, HIV and medication history and body mass index (BMI). Moreover, a 12 h overnight fasting blood sample to determine liver biochemistries, lipid profile, hematological and immuno-virological parameters was drawn within 1 month from the TE examination.

Liver fibrosis was assessed by transient elastography (FibroScan, Echosens, Paris, France) on 4 h fasting individuals by maximum two experienced operators at each site (>100 examinations before the study) [47,54]. The standard M probe was used in all individuals and the XL probe was used in case of failure with M probe or in case of skin to liver distance >2.5 cm, as suggested by the manufacturer [47]. Liver stiffness measurement was expressed in kilopascal (kPa) and calculated as the median value of 10 successful acquisitions, defined by a success rate of at least 60%, and by an interquartile range lower than 30%, as previously described and as suggested by the manufacturing company [47].

Non-invasive serum biomarker liver fibrosis scores (FIB-4 index and APRI scores) were calculated as close as possible to the date of TE [48]. The formula used to calculate APRI score was: (AST/upper limit of normal considered as 40 IU/L)/platelet count (expressed as platelets × 10^9^/L) × 100. FIB-4 index was calculated as: age [years] × AST [IU/L]/platelet count [expressed as platelets × 10^9^/L] × (ALT^1/2^[IU/L]) [49,50].

According to the previous data from literature, we considered consistent with significant liver stiffness measurement values at TE >6.65 kPa (stage F0–F1: 0–6.65 kPa; F2: >6.65–7.9 kPa; F3: >7.9–9.6 kPa; F4: >9.6 kPa), FIB-4 score > 2.67 and APRI score > 1.5. FIB-4 scores < 1.30 and APRI score < 0.5 were considered consistent with low-grade or absent liver fibrosis [51,52,53,54].

Type 2 diabetes mellitus was defined as a fasting blood glucose level ≥126 mg/dL or as treatment with glucose-lowering drugs. According to WHO standards, cut-offs for BMI categories were defined as follows: underweight <18.5 kg/m^2^, normal 18.5–24.9 kg/m^2^, overweight >25 kg/m^2^, and obese ≥30 kg/m^2^. Hyperlipidemia was defined as low-density lipoprotein (LDL) cholesterol levels ≥130 mg/dL and/or total cholesterol ≥200 mg/dL and/or tryglicerides ≥150 mg/dL and/or treatment with lipid-lowering drugs.

CD4 cells count was determined as follows: 50 μL of EDTA-blood cells were processed by flow cytometry using a diagnostic panel of monoclonal antibodies (BD Multitest™ 6-color TBNK, San Jose, CA, USA), including FITC-labeled CD3, PE-labeled CD16 and CD56, PerCPCy5.5-labeled CD45, PECy7-labeled CD4, APC-labeled CD19, and APCCy7-labeled CD8, according to the manufacturer’s instructions. At least 300,000 events were collected for each sample. Data were analyzed using FACSCanto II flow cytometry and CANTO software (BD-Biosciences San Jose, CA, USA).

### 2.3. Statistical Analysis

Continuous variables were summarized as median ± interquartile range (IQR), and categorical variables as frequency and percentage. Predictors of significant liver fibrosis were investigated by unadjusted and adjusted regression models and reported as adjusted odds ratios (aOR) with 95% confidence interval (CI). All adjusted regression models included covariates that were determined a priori to be clinically important, according to previous literature data, or those with *p*-value < 0.05 in univariable analysis.

The correlation between TE/FIB-4 and TE/APRI was evaluated by non-parametric Spearman correlation and kappa statistic.

Statistical analyses were performed using the SPSS Software, version 23.0 (SPSS Inc., Chicago, IL, USA).

## 3. Results

### 3.1. Study Population

We enrolled 234 PLWH who fulfilled the inclusion and exclusion criteria. Table 1 reports the baseline characteristics of the whole population.

Caucasian ethnicity, male gender and sexual intercourse as a risk factor prevailed, respectively, in 89.3%, 67.5%, and 71% of cases. A quarter of the whole population reported a CDC stage C and a nadir CD4 cells count <200 cell/μL. Among 186 individuals on three-drug regimens, 70 (37.6%) were treated with INSTIs, 32 (17.2%) with PIs or b-PIs, 82 (44.1%) with NNRTIs and 2 (1.1%) with other regimens. NRTI backbones were tenofovir disoproxil fumarate (TDF) or tenofovir alafenamide (TAF) + emtricitabine (FTC) in 132 (70.9%) and abacavir (ABC) + lamivudine (3TC) in 54 (29.1%). Among 38 participants on <3-drug regimens, 15 (39.5%) were treated with dolutegravir (DTG) + 3TC, 9 (23.7%) with boosted (b)-PIs monotherapy, 6 (15.8%) with boosted-PIs + 3TC, 5 (13.1%) with b-PIs + INSTIs, and 3 (7.9%) with other combinations. Three individuals were treated with 2NRTIs + INSTI + bPI.

According to previous antiretroviral drug exposure, almost all the population was exposed to NRTIs and more than 50% to NNRTIs and PIs during a median HIV seropositivity time of 11 (IQR 5–17) years.

A previous HBV exposure (HBcAb positivity) was reported in 26% of patients, and metabolic abnormalities were present in 17 (7%) subjects as diabetes mellitus, in 101 (43.2%) as dyslipidemia and in 73 (35.5%) as BMI > 25 kg/m^2^. Aspartate aminotransferase, alanine aminotransferase, total bilirubin and platelet levels tended to be in the normal range.

### 3.2. Prevalence of Liver Fibrosis

The median values of liver stiffness measurements by TE was 4.6 kPa (IQR 4.0–5.8 kPa): F0–F1 stage was observed in 203 participants (86.8%), F2 in 24 (10.2%), F3 in 6 (2.6%) and F4 in 1 case (0.4%).

The median APRI score among 229 PLWH was 0.25 (IQR 0.18–0.31), with APRI < 0.5 in 220 (96.0%) individuals, ≥1.5 in 1 (0.4%) and ≥0.5 but <1.5 in 8 (3.6%).

The median FIB-4 score among 228 PLWH was 0.92 (IQR 0.71–1.20), with FIB-4< 1.3 in 211 (92.3%) patients, ≥2.67 in 3 (1.3%) and ≥1.3 but <2.67 in 14 (6.1%).

### 3.3. Predictors of Liver Fibrosis Assessed by Transient Elastography

In the multivariate linear regression model, after adjustment for potential confounders, a worse liver stiffness (measured by TE) was found to be associated with higher BMI (adjusted mean change +0.11 per 1 unit more; 95% CI + 0.00/ + 0.19; *p* = 0.01), diabetes mellitus (adjusted mean change +2.05; 95% CI + 0.77/ + 3.32; *p* < 0.01), higher baseline HIV-1 RNA detectable (adjusted mean change +1.07; 95% CI + 0.42/ + 1.72; *p* < 0.01) and longer cumulative atazanavir exposure (adjusted mean change +0.14 per 1 year more; 95% CI + 0.05/ + 0.24; *p* < 0.01) (Table 2).

In the multivariate logistic regression model, after adjustment for potential confounders, liver fibrosis ≥F2 (stiffness ≥6.65 kPa) was found to be significantly associated with detectable HIV-1 RNA (vs. undetectable aOR +3.51; 95% CI + 1.47/ + 8.38; *p* < 0.01) and longer cumulative raltegravir exposure (aOR + 1.23 per 1 year more; 95% CI + 1.02/ + 1.17; *p* = 0.02) (Table 3).

### 3.4. Predictors of Liver Fibrosis Assessed by APRI Score

In the multivariate linear regression model, after adjustment for potential confounders, a higher APRI score was found to be associated with CDC C stage (adjusted mean change +0.06; 95% CI + 0.02/ + 0.09; *p* < 0.01) and a longer exposure to tenofovir alafenamide (adjusted mean change +0.03 per 1 year more; 95% CI + 0.01/ + 0.05; *p* < 0.01) (Table 4).

### 3.5. Predictors of Liver Fibrosis Assessed by FIB-4 Score

At multivariate linear regression analysis, after adjustment for potential confounders, we found association between higher FIB-4 scores and HbcAb positivity (adjusted mean change +0.17; 95% CI + 0.01/ + 0.34; *p* = 0.04) and longer cumulative exposure to TAF (adjusted mean change +0.09; 95% CI + 0.03/ + 0.14; *p* < 0.01) (Table 5).

### 3.6. Correlation between Liver Stiffness Measured by TE and APRI or FIB-4 Scores

**Figure 1:**
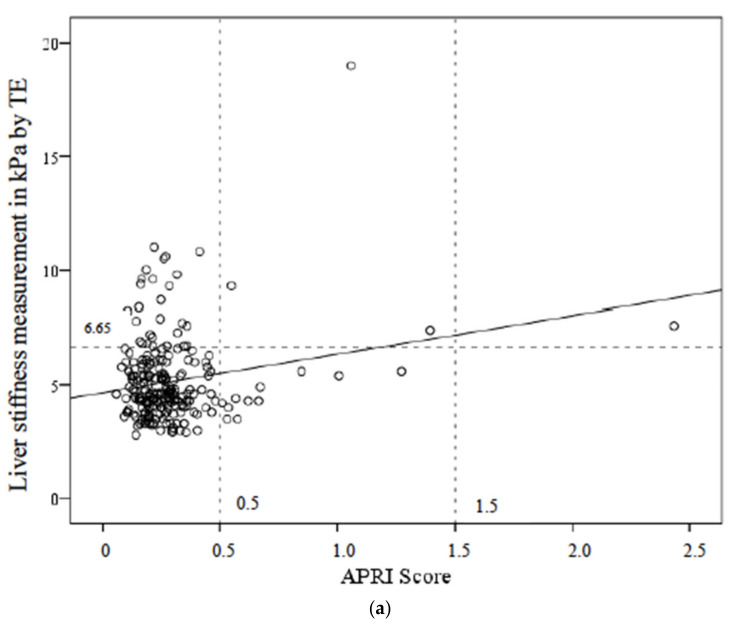

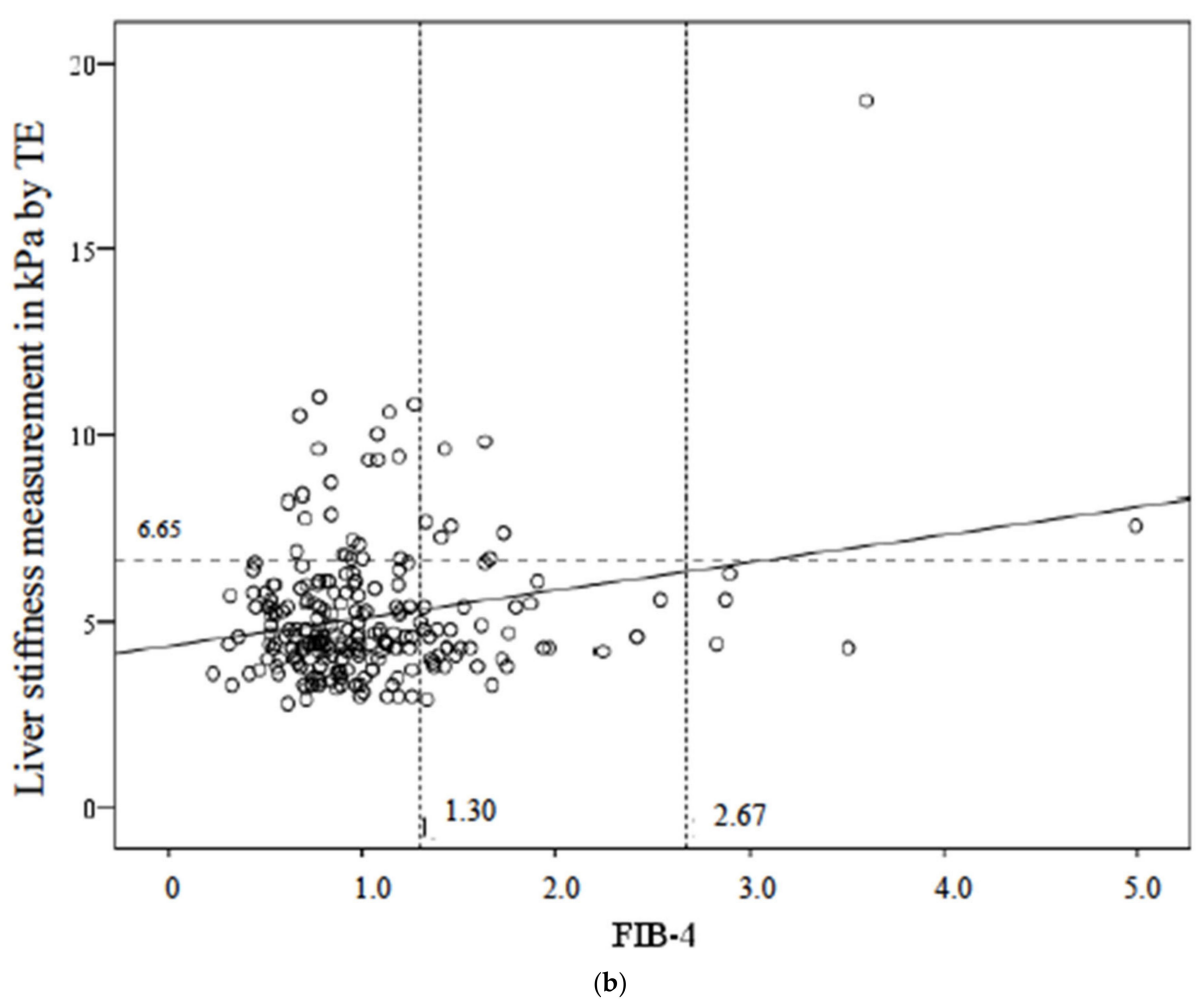
(**a**) Correlation between liver stiffness assessed by TE and APRI score. Rho Spearman 0.006; *p* = 0.93. (**b**) Correlation between livers stiffness assessed by TE and FIB-4 score. Rho Spearman 0.05; *p* = 0.41.

A poor correlation between liver stiffness measured by TE and APRI score (Rho Spearman 0.006; *p* = 0.93) or FIB-4 score (Rho Spearman 0.05; *p* = 0.41) was observed by Spearman correlation test. However, the two models did not reach statistical significance (Figure 1a,b).

### 3.7. Concordance between Significant Liver Stiffness by TE and APRI/FIB-4 Scores

A significant liver fibrosis was defined as a stiffness >6.65 kPa by TE, an APRI score > 1.5, and a FIB-4 score > 2.67. Concordance between liver stiffness measured by TE and higher APRI and FIB4 scores in detecting significant liver fibrosis was studied using Kappa statistics. When comparing the concordance between TE results and APRI score, we observed a Cohen’s K of 0.05 (*p* = 0.01), while Cohen’s K was 0.10 (*p* < 0.01) when comparing TE results and FIB-4 score. Thus, we can infer that the concordance between TE results and APRI score or FIB-4 score in detecting significant liver fibrosis was poor.

## 4. Discussion

Liver disease is one of the major causes of morbidity and mortality in PLWH [17,18]. As far as liver fibrosis is concerned, available studies report that its prevalence in HIV-infected patients ranges from 1.4% to 63% according to different populations [20,28,29,55,56]. Several variables seem to contribute to liver fibrosis in HIV+ patients. Most liver events occur in PLWH co-infected with HBV-HCV viruses or in those with significant alcohol consumption. However, other factors could contribute to evolving liver fibrosis also in HIV infected subjects without viral hepatitis, but limited data are available on the prevalence and predictors of significant liver fibrosis in such population.

In our real-life population of HIV-infected people without Hepatitis Virus infection undergoing assessment of liver fibrosis at three Italian cohorts, the overall prevalence of liver stiffness ≥F2 measured by transient elastography was 13.2%. Importantly, liver biochemistry and platelet levels tended to be in the normal range in a wide proportion of study population. Since such a proportion is not negligible, this finding suggests that assessment of liver fibrosis should also be considered in HIV-infected people without Hepatitis Virus infection, especially in those with metabolic disorders, previous definitive AIDS conditions, past HBV infection, and longer exposure to antiretroviral drugs, particularly to NRTIs and PIs.

We also investigated predictors of liver fibrosis in our population. Similarly to previous studies, higher BMI and diabetes mellitus predicted severe liver fibrosis, suggesting an important role of metabolic disorders in progressive liver damage [22,25]. Globally, non-alcoholic fatty liver disease (NAFLD) is becoming the leading cause of chronic liver diseases [57,58]. PLWH have an increased risk of NAFLD due to chronic inflammation, persistent immune activation, and metabolic disorders [59]. According to a recent meta-analysis including five studies, the estimated prevalence of NAFLD among PLWH is around 35% and its liver-related prognosis is due to the amount of liver fibrosis accumulating over the years [60,61].

Currently available antiretroviral drugs have dramatically changed the landscape of HIV infection, reducing mortality for any reason but increasing the impact of aging-related comorbidities. However, even if many controversies still exist, the potential impact of some antiretroviral drugs in mid- and long-term liver abnormalities is known, in particular due to first-generation NRTIs, such as AZT and PIs [17,38]. In our study, we observed an effect of longer cumulative atazanavir exposure on liver stiffness measurements by TE, according to previous literature data. Strikingly, a higher risk of increased liver fibrosis was associated to longer cumulative TAF exposure according to APRI and FIB-4 scores and to a longer cumulative RAL exposure among those with liver stiffness ≥F2 at TE. These data are in line with previously published data, which showed that both drugs were associated with a significant weight gain after initiation of therapy and with development and progression of liver steatosis [62]. The accumulation of fat can cause liver inflammation, hepatic cell death and steatohepatitis, leading to faster progression of liver fibrosis over time in the absence of an effective intervention for harm reduction. Notably, in our cohort, almost all patients experienced previous NRTIs exposure and therefore TAF exposure could be a proxy for longer NRTI exposure over the years.

Although our study cohort exclusively included PLWH with HIV-1 RNA <50 copies/mL, liver fibrosis at TE was positively correlated with detectable baseline HIV-1 RNA, according to some previous experiences [63]. Despite multiple pathways of liver fibrosis development potentially existing, this interesting finding seems to confirm the association between HIV replication itself and liver fibrosis [64], probably related to an increased hepatic inflammation and fibrogenesis exacerbated by the virus.

Confirming previous findings, we observed an association between higher liver fibrosis predicted by APRI score and CDC C stage, underlining an effect of advanced immune system impairment at presentation [39,63].

Finally, a higher FIB-4 score was associated with HbcAb positivity in our cohort enrolling HBsAg-negative HIV-infected people. Although HBcAb contribution in liver damage in HIV/HCV-infected individuals is well known, recently, other cohort studies seemed to exclude its association with advanced liver fibrosis in HIV infection without HCV [65,66,67]. Generally, HBcAb is considered the sign of previous HBV infection; however, it could be the marker of occult B infection (OBI), a clinical condition defined by the persistence of intrahepatic cccHBV-DNA. The risk of HBV-DNA replication/reactivation in OBI strongly supports the need of a careful monitoring for sign of past HBV infection [68].

In clinical practice, non-invasive methods are widely used to estimate of liver fibrosis. The non-invasive methods could identify PLWH at higher risk of severe liver injury, and liver fibrosis stratification may help to determine those who may benefit from lifestyle changes, including healthy diet, weight loss, physical exercise and metabolic disorder control, and potential pharmacologic interventions [17,18,20]. Non-invasive assessment of liver fibrosis can be performed (i) by biological approaches, quantifying serum biomarkers, with FIB-4 and APRI being the most used scores, or (ii) by a physical approach, measuring liver stiffness by TE. However, only a few non-invasive methods are validated in the specific setting of HIV mono-infection, and most data are available in adults with elevated aminotransferases on antiretroviral therapy [69].

Interestingly, in our population, mainly constituted by HIV-infected patients without hepatitis virus infection with normal aminotransferase levels, the different investigated non-invasive methods (transient elastography, FIB-4, and APRI) were discordant to predict liver fibrosis in this setting.

According to previous studies, a poor concordance between serum fibrosis biomarkers and TE results in detecting severe liver fibrosis in the specific setting of HIV monoinfection [70]. FIB-4 index was deeply influenced by age, whereas TE and APRI score were not. TE had the advantages over FIB-4 index and APRI score to be associated with portal hypertension and to be more accurate to detect advanced fibrosis in people affected by non-alcoholic fatty liver disease [71].

The strengths of the study are the large population recruited from three distinct clinical units in northern and central Italy and the use of easily accessible and validated non-invasive instrumental and labs tool to investigate liver fibrosis.

The main limitations include the inter-operator and instrument variability for transient elastography liver stiffness measurement and the lack of histological data to compare non-invasive methods results. Liver biopsy is considered the gold standard for the diagnosis of liver fibrosis [72]. However, this invasive procedure is only suitable for a selected group of people because of the risk of complications.

Indeed, given the lack of data regarding waist circumference to characterize participants phenotype, BMI was used to categorize normal and overweight/obese people, even if the potential underestimation of obesity by using only BMI in people with abnormal fat distribution is known (high visceral adiposity and/or low muscle mass).

## 5. Conclusions

In virologically suppressed HIV-1-infected people without viral hepatitis, we observed a significant liver fibrosis in a non-negligible proportion of subjects (13.2%). Besides classic metabolic factors, some HIV-related variables (detectable HIV-RNA, exposure to certain antiretroviral drugs, previous AIDS events) were associated with an increased risk of significant fibrosis, suggesting that this population could be at increased risk of liver disease. Indeed, early detection of liver fibrosis is extremely important due to its independent risk of progression to severe liver damage, especially in PLWH with metabolic disorders, advanced HIV presentation and longer antiretroviral drug exposure.

Our findings also demonstrated that the different investigated non-invasive methods (transient elastography, FIB-4, and APRI) were poorly concordant to predict severe liver fibrosis in this setting. As a consequence, the best method for the estimation of liver fibrosis in this setting remains to be determined. A larger cohort, including more people with severe liver fibrosis, could offer the opportunity to explore different cut-off to improve sensibility and sensitivity of investigated methods.

Further longitudinal studies are needed because PLWH need a better understanding of liver disease beyond viral hepatitis coinfection.

## Figures and Tables

**Table 1:** Baseline characteristics (*n* = 234).

Age, Years ^a^	49 (42−56)
Gender, male ^b^	158 (67.5)
Ethnicity ^b^	
Caucasian	209 (89.3)
African	18 (7.7)
Asian	5 (2.1)
Hispanic	2 (0.9)
Risk factors for HIV ^b^	
Sexual intercourses	166 (71.0)
Males who have sex with males	69 (29.5)
Heterosexuals	97 (41.5)
Injecting drug users	42 (17.9)
Other/unknown	26 (11.1)
Time from HIV diagnosis, years ^a^	11 (5−17)
Nadir CD4 cells count, cell/Μl ^a^ (*n* = 211)	255 (127−485)
Nadir CD4/CD8 ^a^ (*n* = 106)	0.38 (0.18−0.67)
Zenith HIV-1 RNA, log_10_ cp/mL ^a^ (*n* = 141)	4.84 (4.33−5.43)
CDC stage C ^b^ (*n* = 231)	60 (25.7)
Baseline CD4 cells count, cell/μL ^a^ (*n* = 233)	670 (515−853)
Baseline CD4/CD8 ^a^ (*n* = 228)	0.9 (0.6−1.4)
Baseline HIV-1 RNA <50 cp/mL but detectable ^a^	67 (28.6)
Current antiretroviral therapy ^b^	
3 Drug regimens	186 (79.1)
<3 Drug regimens	45 (19.6)
>3 Drug regimens	3 (1.3)
Previous antiretroviral drugs used ^b^	
NRTI	233 (99.6)
PI or bPI	153 (65.4)
NNRTI	137 (58.5)
INSTI	106 (45.3)
MVC	9 (3.8)
T20	6 (2.6)
Current antiretroviral regimens ^b^	
INSTI-based	88 (37.6)
NNRTI-based	84 (35.9)
PI or bPI-based	49 (20.9)
Other	13 (5.5)
HBcAb positivity ^b^	62 (26.5)
Alcohol user ^b^	70 (29.9)
Smokers ^b^	85 (36.3)
Diabetes mellitus ^b^	17 (7.3)
Glycemia, mg/dL ^a^ (*n* = 228)	88 (82.2−96.7)
Dyslipidemia ^b^	164 (70.0)
Total cholesterol, mg/dL ^a^ (*n* = 230)	201 (180−227)
LDL cholesterol, mg/dL ^a^ (*n* = 191)	130 (108−154)
Triglycerides, mg/dL ^a^ (*n* = 230)	113 (80−165)
Aspartate aminotransferase, IU/mL ^a^ (*n* = 230)	21 (17−26)
Alanine aminotransferase, IU/mL ^a^ (*n* = 230)	21 (15−30)
Platelets, 10^3^/mmc ^a^ (*n* = 230)	229 (197−268)
Total bilirubin, mg/dL ^a^ (*n* = 214)	0.4 (0.3−0.6)
Gamma-glutamyl transferase, U/L ^a^ (*n* = 231)	24 (16−40)
BMI ^a^ (*n* = 233)	23.7 (21.4−26.5)
Underweight ^b^ < 18.5 kg/m^2^	13 (5.6)
Normal weight ^b^ 18.5–24.9 kg/m^2^	137 (58.5)
Overweight ^b^ > 25 kg/m^2^	68 (29.1)
Obesity ^b^ ≥ 30 kg/m^2^	15 (6.4)
Median stiffness at TE, in kPa ^a^	4.6 (4.0−5.8)
Fibrosis stage at TE ^b^	
F0–F1	203 (86.8)
F2	24 (10.2)
F3	6 (2.6)
F4	1 (0.4)
Median APRI score ^a^ (*n* = 229)	0.25 (0.18−0.31)
APRI < 0.5 ^b^	220 (96.1)
0.5 ≤ APRI < 1.5 ^b^	8 (3.5)
APRI ≥ 1.5 ^b^	1 (0.4)
Median FIB-4 score ^a^ (*n* = 228)	0.92 (0.71−1.20)
FIB-4 < 1.3 ^b^	211 (92.5)
1.3 ≤ FIB-4 < 2.67 ^b^	14 (6.1)
FIB-4 ≥ 2.67 ^b^	3 (1.3)

^a^ Median (IQR); ^b^ n (%); unless otherwise indicated. Table 1 legend: APRI, AST-to-platelet ratio index; BMI, body mass index; CDC, United States Centers for Disease Control and Prevention; FIB-4, fibrosis-4 score; HIV, human immunodeficiency virus; INSTI, integrase strand transfer inhibitor; LDL cholesterol, low-density lipoprotein; MVC, maraviroc; NNRTI, non-nucleoside reverse-transcriptase inhibitor; NRTI, nucleos(t)ide reverse-transcriptase inhibitor; PI, protease inhibitor; bPI, boosted protease inhibitor; T20, enfuvirtide; TE, transient elastography.

**Table 2:** Factors associated with liver stiffness (measured by TE) by linear regression models.

Univariate Analysis				Multivariate Analysis		
Variable	Mean Change	95% CI	*p*-Value	Adjusted Mean Change	95% CI	*p*-Value
Age, per 10 years increase	0.40	0.10/0.41	<0.01	0.24	−0.09/0.56	0.15
Male gender	0.65	0.15/1.15	0.01	−0.81	−1.63/0.01	0.05
BMI, per 1 unit more	0.10	0.04/0.16	<0.01	0.11	0.00/0.19	**0.01**
Diabetes mellitus	1.60	0.71/2.49	<0.01	2.05	0.77/3.32	**<0.01**
Time from HIV diagnosis, per 1 year more	0.05	0.02/0.07	<0.01	0.01	−0.06/0.05	0.96
Nadir CD4, per 100 cell/μL increase	−0.26	−0.49/0.04	0.02	−0.13	−0.35/0.08	0.21
Baseline CD4/CD8	−0.38	−0.76/0.01	0.05	−0.30	−0.79/0.18	0.22
Detectable baseline HIV-1 RNA	1.27	0.77/1.77	<0.01	1.07	0.42/1.72	**<0.01**
Cumulative AZT exposure, per 1 year more	0.06	0.01/0.16	0.04	−0.04	−0.13/0.06	0.42
Cumulative ATV exposure, per 1 year more	0.08	0.01/0.16	0.02	0.14	0.05/0.24	**<0.01**

Legend: ATV, atazanavir; AZT, azidothymidine; BMI, body mass index.

**Table 3:** Predictors of liver fibrosis ≥ F2 (measured by TE) by logistic regression models.

Univariate Analysis				Multivariate Analysis		
Variable	OR	95% CI	*p*-Value	aOR	95% CI	*p*-Value
Age, per 10 years increase	1.94	1.32/2.84	<0.01	1.48	0.95/2.32	0.08
Alcohol consumption *	0.31	0.10/0.91	0.03	0.35	0.11/1.17	0.09
Years of HIV, per 1 year more	1.09	1.04/1.14	<0.01	1.03	0.96/1.10	0.38
Diabetes mellitus	4.19	1.42/12.32	<0.01	2.19	0.59/8.10	0.24
Detectable baseline HIV-1 RNA	4.35	1.99/9.52	<0.01	3.51	1.47/8.38	**<0.01**
Cumulative AZT exposure, per 1 year more	1.19	1.03/1.19	<0.01	1.07	0.97/1.19	0.19
Cumulative ATV exposure, per 1 year more	1.13	1.03/1.24	<0.01	1.10	0.99/1.24	0.08
Cumulative RAL exposure, per 1 year more	1.24	1.06/1.45	<0.01	1.23	1.02/1.17	**0.02**

Legend: ATV, atazanavir; AZT, azidothymidine; BMI, body mass index; RAL, raltegravir. * Alcohol consumption: not significant alcohol intake, defined as daily consumption less than 30 g for males and 20 g for females.

**Table 4:** Factors associated with APRI score by linear regression models.

Univariate Analysis				Multivariate Analysis		
Variable	Mean Change	95% CI	*p*-Value	Adjusted Mean Change	95% CI	*p*-Value
CDC C stage	0.08	0.05/0.11	<0.01	0.06	0.02/0.09	**<0.01**
HbcAb positivity	0.07	0.00/0.13	0.04	0.05	−0.01/0.11	0.11
Diabetes mellitus	0.16	0.05/0.27	<0.01	0.08	−0.02/0.18	0.21
Cumulative D4T, per 1 year more	0.02	0.01/0.04	<0.01	0.01	0.00/ 0.02	0.14
Cumulative TAF, per 1 year more	0.05	0.03/0.06	<0.01	0.03	0.01/0.05	**<0.01**

Legend: D4T, stavudine; TAF, tenofovir alafenamide.

**Table 5:** Predictors of liver fibrosis assessed by FIB-4 score by linear regression models.

Univariate Analysis				Multivariate Analysis		
Variable	Mean Change	95% CI	*p*-Value	Adjusted Mean Change	95% CI	*p*-Value
Time from HIV diagnosis, per 1 year more	0.01	0.01/0.02	<0.01	0.00	−0.01/0.01	0.78
HbcAb positivity	0.20	0.04/0.37	0.02	0.17	0.01/0.34	**0.04**
Diabetes mellitus	0.38	0.10/0.65	<0.01	0.21	−0.07/0.49	0.15
Osteoporosis	0.28	0.01/0.55	0.04	0.12	−0.15/0.39	0.40
Cumulative AZT exposure, per 1 year more	0.02	−0.00/0.03	0.06	−0.01	−0.03/0.01	0.45
Cumulative ddC exposure, per 1 year more	0.13	0.01/0.25	0.04	0.10	−0.02/0.22	0.10
Cumulative D4T exposure, per 1 year more	0.05	0.01/0.09	<0.01	0.03	−0.02/0.07	0.21
Cumulative 3TC exposure, per 1 year more	0.02	0.00/0.03	0.02	0.01	−0.01/0.03	0.23

Legend: AZT, azidothymidine; ddC, zalcitabine; D4T, stavudine; TAF, tenofovir alafenamide.

## Data Availability

Not applicable.
